# A Rare Familial Case of Harlequin Ichthyosis in an Infant of a Diabetic Mother: A Diagnostic and Management Challenge in Low and Middle Income Settings

**DOI:** 10.1002/ccr3.71371

**Published:** 2025-10-27

**Authors:** Muhammad Zaeem, Ameer Hamza Mehmood Ul Hassan, Muhammad Hassaan Javaid, Muhammad Usman, Muhammad Ahmed, Muhammad Usama Naveed, Mohammad Yassin Al Aboud

**Affiliations:** ^1^ Institution Rawalpindi Medical University Rawalpindi Pakistan; ^2^ Institution Shalamar Institute of Health Sciences Lahore Pakistan; ^3^ Institution Shifa College of Medicine Islamabad Pakistan; ^4^ Institution Nishtar Medical University Multan Pakistan; ^5^ Institution Faculty of Medicine Latakia University Latakia Syria

**Keywords:** consanguinity, harlequin ichthyosis, LMICs, maternal diabetes, neonatal sepsis

## Abstract

Harlequin Ichthyosis (HI) is an extremely rare, autosomal recessive, and highly fatal condition in neonates. It is especially difficult to control in the low‐ and middle‐income countries (LMICs) due to the low rate of prenatal screening, cultural reluctance, and lack of access to neonatal intensive care. We present a 37‐week neonate born to a diabetic mother from a consanguineous marriage with a background of HI‐related neonatal mortality. The child had classical manifestations of HI in the form of hyperkeratotic armor‐like plates, eclabium, ectropion, and limb contractures. Oral acitretin was started at 1.1 mg/kg/day along with intensive supportive care but the neonate had become septic and succumbed on day nine despite multi‐antibiotic therapy, intravenous fluid resuscitation, and oral retinoid therapy. There was mild dermatological improvement with retinoid therapy but systemic improvement was not observed. Antenatal diagnosis was not made because of poor prenatal care and religious reluctance. On record, the present case is the first reported one for our region showing a likely association between maternal diabetes and high HI severity because epigenetic studies suggest that hyperglycemia may alter fetal gene expression, particularly in pathways related to cell adhesion and barrier function. The case highlights the intersection of maternal comorbidities, consanguinity, and healthcare system gaps in aggravating HI outcomes in LMICs. It necessitates emergent interventions such as subsidized prenatal screening, genetic counseling outreach, particularly concerning maternal diabetes, additional research, consanguineous marriage counseling, and neonatal infection control to enhance prognosis and prevent recurrence.

**Summary**

- Neonatal harlequin Ichthyosis has important diagnostic and management consequences in low‐resource environments.
- The situation is further complicated by familial recurrence and maternal diabetes and considers the potential that maternal comorbidities can act as a prognostic indicator and underscores the value of prenatal genetic counseling, screening, and enhanced perinatal infection control in low‐ and middle‐income countries.

## 1 Introduction

Harlequin Ichthyosis, also known as keratosis diffusa fetalis, is a rare autosomal recessive dermatological disorder affecting both sexes equally (1:1) [1]. Characterized by hard thickened armor‐like plates of skin covering the entire body, which resemble a harlequin costume, it results from mutations in the ABCA 12 gene. HI has an incidence rate of 1 in 300,000 births [2]. The mortality rate of HI is very high worldwide.

No doubt, the mortality rate is improving worldwide with the availability of better treatment options and improved preventive measures for infectious diseases; however, the condition is still a major concern for underdeveloped countries [3]. HI is generally devastating in low‐ and middle‐income countries (LMICs), where genetic testing may not be available, neonatal support and prenatal diagnosis may be limited, and sociocultural stigma and gaps in health system infrastructure, such as in sub‐optimal Neonatal Intensive Care Units (NICUs) add to the difficulties [4]. As the disease is genetic in nature, effective genetic counseling and prenatal diagnosis are a major part of management to prevent the psychological burden of pregnancy as well as emotional trauma for neonatal mortality [5].

Along with many other factors, the religious impact on these disorders is very large. Previous studies have proven that the evasive response of parents toward prenatal checkups and diagnosis plays a crucial role. Studies in Muslim majority countries like Pakistan and Lebanon have shown that religious convictions are a primary reason for refusing prenatal testing and termination [6, 7].

With the literature, it is evident that gestational diabetes increases the neonatal ICU stay, along with many other fetal complications. However, its impact on a rare diagnosis such as Harlequin Ichthyosis requires further investigation [8].

Therefore, we hypothesize that maternal diabetes may enhance the severity or prognosis of HI. This case reports a rare and interesting case of a neonate with harlequin ichthyosis in a mother with diabetes along with a family history of prior similar mortality. This case not only highlights the unique clinical presentation of the patient but also the diagnostic challenges stemming from financial and religious concerns and the lack of proper management due to the rarity and familial nature of this case in a struggling healthcare system.

## 2 Case Presentation

A 30‐year‐old multiparous lady gravida 5 para 4 with a history of diabetes mellitus type 2, gestational hypertension, and an obstetric surgical history of previously 4 cesarean deliveries reported to the Labor Room of our Hospital for her 5th cesarean delivery at 37 weeks of gestation based on ultrasound dating. Out of her 4 previous cesarean deliveries one resulted in an abortion due to tubal ectopic pregnancy. She had one premature delivery and the baby died due to respiratory distress syndrome. She also had one prior delivery with harlequin ichthyosis and the baby subsequently died due to hospital‐acquired pneumonia, while the last one resulted in the birth of a normal healthy girl. The parents have been in a consanguineous marriage for around 12 years. She had a drug history of metformin for her diabetes. She also reported inconsistent adherence to prescribed medication and her glycemic control was suboptimal (HbA1c 10.6). Her menstrual cycles were normal. The patient had no past family history of any disease.

### 2.1 Clinical Findings

At the time of birth, the general physical examination was done. Upon inspection the baby had clinical manifestations that led to the likely diagnosis of Harlequin Ichthyosis and the patient's prior history also confirmed it.

**FIGURE 1:**
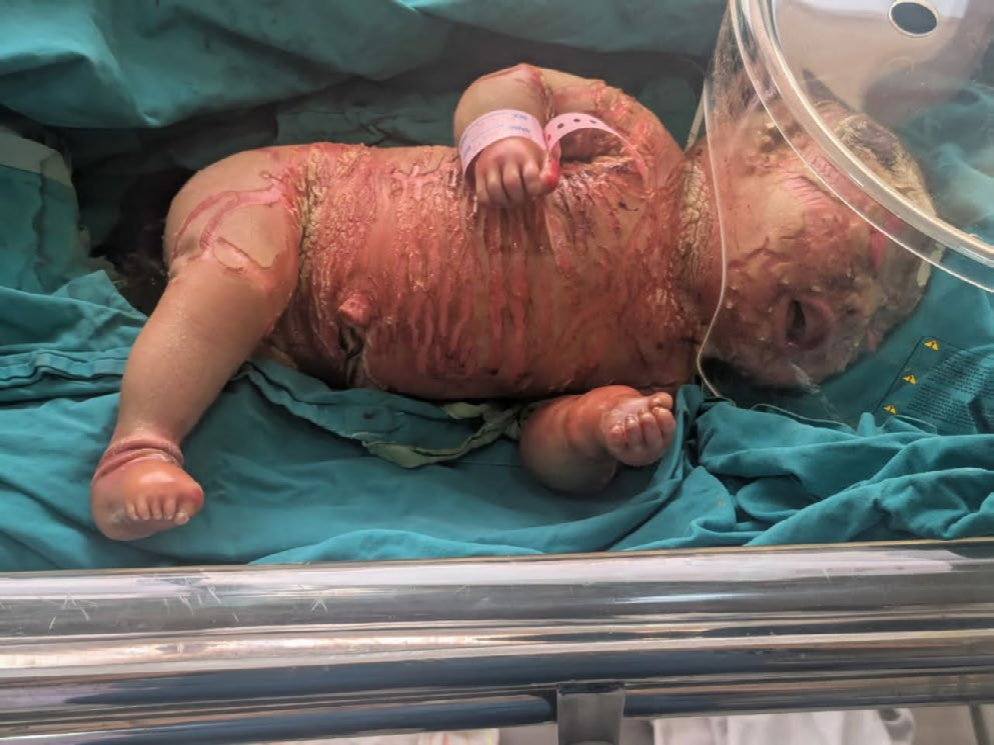
A neonate suffering from Harlequin Ichthyosis.

The neonate had dry, scaly skin all over the body giving the appearance of yellowish‐gray armor‐like plates of thick skin separated by deep fissures, eclabium, hypoplastic ears and nose, contractures of phalanges, and flat anterior and posterior fontanelles, as well as bilateral ectropion. The digits were hypoplastic and palmar and plantar surfaces were erythematous. There was partial hair loss on the scalp (alopecia). There were no signs of pallor, jaundice and cyanosis (Figure 1). The baby had an APGAR score of about 8 at 1 min and 9 in 5 min.

### 2.2 Diagnostic Assessment

The gestational age of the neonate was assessed by the Ballard scoring system and was estimated to be 37 weeks.

Anthropometric measurements at birth revealed a birth weight of 2.7 kg and a body length of 45.8 cm; both were within the lower percentile for gestational age. The neonate's vitals were also assessed. Vitals were normal at the time of birth but over the course of 2 days in NICU, the neonate developed severe respiratory tract infection and was hypotensive with a blood pressure of 60/40 mmHg, heart rate of 175 beats per minute (Tachycardia), and respiratory rate of 72 breaths per minute (tachypnea). The core body temperature was 35°C (Hypothermia). Blood cultures could not be performed because venous access was impeded by the neonate's thickened skin. We tried to take blood samples from the umbilical cord but it was not possible due to backflow of blood. Genetic testing for ABCA12 mutations was not available at our institution.

Antenatal diagnosis was not possible in this case, as the mother presented directly at the time of delivery without any prior antenatal care or investigations. Clinically, the neonate exhibited hallmark features suggestive of Harlequin Ichthyosis, in conjunction with prior family history of HI. Based on the clinical presentation and previous family history a working diagnosis of Harlequin Ichthyosis was established. Differential diagnoses considered included lamellar ichthyosis, and epidermolytic ichthyosis. These were ruled out based on the severity of hyperkeratosis, presence of eclabium/ectropion, and characteristic armor‐like skin plates consistent with HI (As shown in Figure 1.)

### 2.3 Management

Immediately after birth the neonate was transferred to the NICU. The condition was managed appropriately with intravenous fluids (5% dextrose in 60 mL saline solution three times a day) through the umbilical cord to maintain hydration and electrolytes. Fluid output and input were also meticulously monitored to maintain optimal fluid balance. For dermatological treatment, retinoid therapy with acitretin was initiated. A 25 mg acitretin tablet was crushed and dissolved in 25 mL of normal saline. 3 mL (3 cc) of this suspension was administered once a day. The dose was adjusted according to the baby's weight which was about 1.1 mg/kg/day. No acute systemic side effects were observed during therapy. Topical emollients and moisturizers were regularly applied to maintain skin hydration and provide a protective barrier against infection and trans‐epidermal water loss as well as other infection prevention measures (sterile handling, isolation in a warm incubator) were also done to prevent infection. Empirical antibiotic (ampicillin) was commenced prophylactically and later the regimen was modified to add amikacin and vancomycin to combat respiratory tract infection.

### 2.4 Outcomes

Initially the newborn showed gradual improvement because of oral retinoid therapy, antibiotics and necessary measures to restore blood volume, However, the newborn acquired an acute respiratory tract infection which progressed to neonatal sepsis. Despite all efforts and intensive care, the baby's condition began to deteriorate. Tragically the patient succumbed to death on the ninth day of life.

## 3 Discussion

Harlequin Ichthyosis is one of the most life‐threatening skin disorders with particularly difficult management challenges in LMICs where advanced neonatal and dermatological care is scarce. The major challenges arise from inadequate prenatal diagnostic services, restricted access to essential treatments, and insufficient neonatal care infrastructure, all of which exacerbate poor outcomes in developing countries [4].

As evident from previous studies these antenatal care challenges in our country arise from a lack of awareness among our community, access issues and inadequate community support along with gender discrimination and income inequalities [9, 10].

According to the most recent data from 2025, currently only 200 cases of HI have been reported in medical literature worldwide. The hallmark features observed in our patient like thickened hyperkeratotic plates, ectropion, and eclabium were consistent with the phenotypic spectrum previously reported in literature [11]. However, due to the far lesser number of cases present it is difficult to pinpoint all the factors part of its causation apart from the genetic makeup. Our study therefore focuses on the possibility of maternal diabetes affecting the prognosis of the subject.

Research has determined that maternal diabetes, both pregestational and gestational, poses significant risks to fetal development and neonatal outcomes along with cardiovascular, neural tube and metabolic complications [12].

Additionally, maternal diabetes has been linked to the expression of genes involved in cell adhesion molecules, which are major determinants in the development of congenital heart diseases in infants [13]. Our case demonstrates the convergence of multiple poor prognostic factors: familial recurrence associated with consanguinity, absence of prenatal care, and maternal type 2 diabetes mellitus. While consanguinity is a well‐established risk factor due to the autosomal recessive inheritance of HI [14], the potential role of maternal diabetes is less understood. Diabetes in pregnancy is strongly associated with congenital anomalies, metabolic instability, and adverse neonatal outcomes [15]. Epigenetic studies suggest that hyperglycemia may alter fetal gene expression, particularly in pathways related to cell adhesion and barrier function Epigenetic changes occur through histone modification, DNA methylation, and disrupted function of noncoding ribonucleic acid (ncRNA), including microRNAs (miRNAs) [16], raising the hypothesis that maternal diabetes could exacerbate phenotypic severity in rare genetic disorders such as HI. Although our observation is intriguing, it remains speculative and warrants validation through larger case series or registry data. Based on this conundrum, we suggest that the link of maternal diabetes to the outcome of Harlequin Ichthyosis should be further explored.

Despite early postnatal interventions like emollients, hydration, systemic antibiotics, and oral retinoid therapy, the neonate succumbed to HI on the 9th day of life. The poor prognosis in our case could be linked to neonatal sepsis. Previous studies have revealed increased mortality in harlequin ichthyosis associated with neonatal sepsis [17]. Our subject validates the outcomes of this study.

Systemic retinoid therapy has been reported to accelerate skin shedding and improve barrier function [18]. A study found that early systemic retinoids aided in the shedding of hyperkeratotic scales in a comprehensive case series of 45 patients, with an overall survival rate of more than 50% [19]. In our patient, acitretin was administered at 1.1 mg/kg/day, resulting in mild dermatological improvement but no systemic benefit. The lack of survival advantage in this case may reflect both the severity of systemic complications and the delay in initiating therapy. Robust supportive neonatal care and effective infection control remain more decisive for survival than pharmacologic therapy alone, which is also a reason for high mortality despite the use of only systemic retinoids Sturrock et al. [20].

Due to emerging evidence regarding the use of retinoids to accelerate skin shedding and enhance skin integrity, Acitretin was given to neonates. Even though the use of systemic retinoids has always been controversial [21, 22].

Our study was not able to strongly suggest the use of systemic steroids as the subject did not survive and no systemic improvements were observed.

Our case also had familial recurrence of HI showing an autosomal recessive inheritance pattern associated with consanguinity. This is in accordance with previous studies which have shown similar associations [23, 24].

Comparative data further highlight the survival disparity between LMIC and high‐income settings. In high‐income settings, survival rates for HI are low with patients surviving to adolescence as exemplified by a case report from the United Kingdom [25]. In contrast the mortality is still high in LMICs due to a lack of proper management as exemplified by a case image from Syria [26]. This disparity reflects not only differences in healthcare infrastructure but also gaps in public health systems, particularly the lack of routine genetic counseling and antenatal screening programs in LMICs.

This reflects a dire need for genetic counseling in regions where consanguineous marriage is prevalent. In well‐developed countries, molecular genetic testing and proper genetic counseling have drastically reduced the recurrence of HI in high‐risk populations [27, 28]. But such facilities are scarcely available in LMICs requiring revised government policies and focused educational activities for the community.

## 4 Conclusion

Harlequin Ichthyosis remains a devastating congenital disorder, especially in low‐resource settings where diagnostic, therapeutic, and genetic services are limited. Our case underscores the multifaceted challenges associated with its management in LMICs, including inadequate prenatal care, limited neonatal intensive support, and absence of accessible genetic counseling. The potential influence of maternal diabetes on disease prognosis, as suggested in this case, opens new avenues for research into modifiable maternal risk factors. Moreover, the familial recurrence observed further reinforces the critical need for integrating genetic screening and counseling into routine antenatal care, particularly in communities with high rates of consanguinity. In light of poor outcomes despite early interventions, a multi‐pronged approach involving policy‐level reforms like subsidizing prenatal screening and genetic testing in high‐risk populations, enhanced culturally sensitive community education campaigns to overcome cultural and religious barriers and infrastructural development is essential to improve prognosis and prevent recurrence of such life‐threatening disorders. Finally, the speculative association between maternal diabetes and HI severity calls for larger, multicenter studies to explore modifiable maternal risk factors in rare genetic disorders.

## Author Contributions

**Muhammad Zaeem:** conceptualization, investigation, methodology, resources. **Ameer Hamza Mehmood Ul Hassan:** resources, writing – original draft, writing – review and editing. **Muhammad Hassaan Javaid:** writing – original draft. **Muhammad Usman:** conceptualization, investigation, writing – review and editing. **Muhammad Ahmed:** writing – review and editing. **Muhammad Usama Naveed:** writing – review and editing. **Mohammad Yassin Al Aboud:** writing – review and editing.

## Consent

Written informed consent was taken from the patient's guardian.

## Conflicts of Interest

The authors declare no conflicts of interest.

## Data Availability

The authors have nothing to report.
